# Advanced UPLC-MS/MS Method for the Quantification of SIPI6398 in Rat Plasma and Its Pharmacokinetic Characterization

**DOI:** 10.1155/2024/9811466

**Published:** 2024-05-06

**Authors:** Fan Chen, Shunjun Ma, Runrun Wang, Dizhong Chen, Congcong Wen, Xianqin Wang, Tao Hu, Xiuwei Shen

**Affiliations:** ^1^Ruian People's Hospital, The Third Affiliated Hospital of Wenzhou Medical University, Wenzhou, China; ^2^Laboratory Animal Centre, Wenzhou Medical University, Wenzhou, China; ^3^School of Pharmaceutical Sciences, Wenzhou Medical University, Wenzhou, China; ^4^Department of Thoracic Surgery, The Affiliated Yantai Yuhuangding Hospital of Qingdao University, Yantai, China

## Abstract

SIPI6398 is a novel anti-schizophrenia agent with a new mechanism of action and demonstrates better target selectivity and safety compared to its competitors. However, few in vivo studies on the pharmacokinetics and bioavailability of SIPI6398 have been performed. A rapid and simple ultra-performance liquid chromatography-tandem mass spectrometry (UPLC-MS/MS) approach was developed for accurate quantification of SIPI6398 in rat plasma. A simple protein precipitation of acetonitrile-methanol (9 : 1, v/v) was used to treat plasma. Chromatography was performed on a UPLC HSS T3 column (50 mm × 2.1 mm, 1.8 *μ*m) at a flow rate of 0.4 ml/min. The mobile phase consisted of acetonitrile-water (with 0.1% formic acid) and gradient elution was used, and the elution time was 4 minutes. Quantitative analysis was performed using electrospray ionization (ESI) in positive ion detection mode with multiple reaction monitoring (MRM) mode. To evaluate the pharmacokinetics and bioavailability, SIPI6398 was administered to rats in two different ways: oral (4 mg/kg) and intravenous (2 mg/kg) administration. The calibration curve for the UPLC-MS/MS approach shows excellent linearity in the range of 1–2000 ng/mL with an *r* value above 0.99. The precision, accuracy, recovery, matrix effect, and stability results all meet the criteria established for biological analytical methods. The UPLC-MS/MS method was successfully applied it to pharmacokinetics study of SIPI6398. The bioavailability of SIPI6398 was calculated to be 13.2%. These studies have the potential to contribute towards a more comprehensive comprehension of the pharmacokinetics and bioavailability of SIPI6398.

## 1. Introduction

Schizophrenia significantly affects human health and is an important cause of disability in patients with mental illness, with a disability rate of 2.8% [1, 2]. The global incidence of schizophrenia is approximately 1%, which represents a significant burden for both patients and their families [3, 4]. Therefore, overcoming schizophrenia, the first “cause of death” of mental illness, is not only a global mental health challenge but also a concern for the entire society. It is also a responsibility and challenge that medical professionals must face. Schizophrenia is a serious mental illness characterized mainly by positive symptoms, negative symptoms, and cognitive deficits [5, 6]. While anti-schizophrenia medications currently on the market can relieve positive and negative symptoms, they cannot effectively combat cognitive impairment, often leading to more serious side effects.

SIPI6398, chemically N-(trans-4-(2-(4-(benzo[d]isothiazol-3-yl)piperazin-1-yl) ethyl)cyclohexyl)furan-2-carboxamide [7], is a new anti-Schizophrenia drug. The innovative drug received clinical trial approval from the National Medical Products Administration (NMPA) in November 2020 [8]. The approval underscores the drug's potential to confer substantial therapeutic benefits in the management of diverse ailments. The novel active multitarget molecule SIPI6398 exhibits a new mechanism of action, effectively ameliorating both positive and negative symptoms of schizophrenia while significantly enhancing cognitive dysfunction. It has the value of further development as a new drug candidate against schizophrenia.

Pharmacokinetics involves the application of kinetic principles and mathematical models to quantitatively describe and summarize the absorption, distribution, metabolism, and excretion (ADME) of drugs administered via different routes (e.g., intravenous injection, intravenous infusion, and oral administration) [9, 10]. Pharmacokinetic testing is an essential process in drug research for new drugs, improved formulations, or off-patent generics. Therefore, to comprehensively understand the in vivo dynamic changes of SIPI6398, it is imperative to investigate its mechanism of action.

The in vivo pharmacokinetics of SIPI6398 have been previously reported [7]; however, the LC-MS/MS method used was not fully validated. The primary objective of this study is to develop a highly accurate UPLC-MS/MS detection method for quantifying the concentration of SIPI6398 in rat plasma. By comprehensively investigating drug concentrations in blood and understanding the pharmacokinetic principles, a better comprehension of the mechanism of action for novel drugs within the body can be achieved. Furthermore, by calculating absolute bioavailability, more precise reference data can be provided for the development and application of new pharmaceuticals.

## 2. Experimental

### 2.1. Reagents

**Figure 1:**
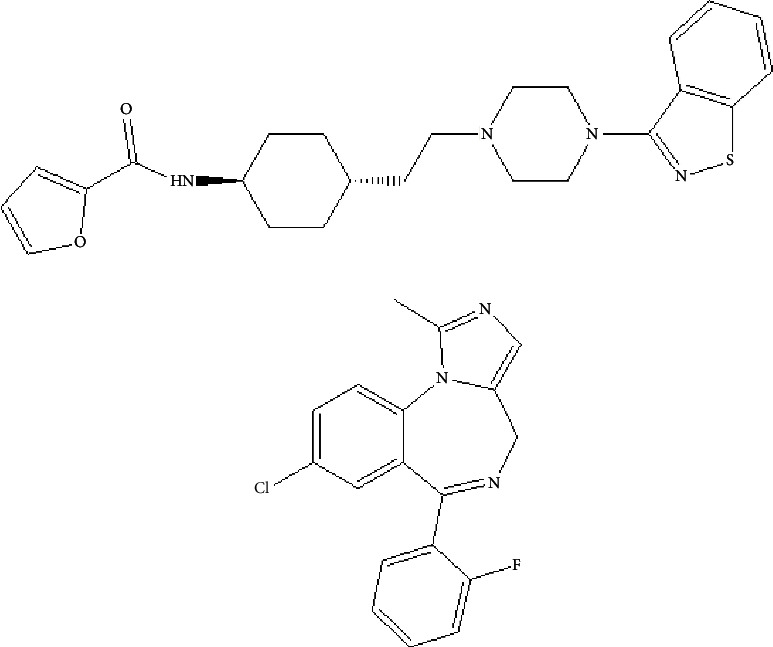
Chemical structure of SIPI6398 (a) and IS (b).

SIPI6398 (purity ≥98%, Figure 1) was obtained from the School of Pharmaceutical Sciences, Wenzhou Medical University (Wenzhou, China). Midazolam (internal standard, IS, purity ≥98%, Figure 1) was purchased from the National Institutes for Food and Drug Control (Beijing, China). Acetonitrile and methanol (chromatographically pure) were provided by Merck Ltd (Darmstadt, Germany). The experimental water was ultrapure water (resistance >18 mΩ) prepared with a Milli-Q purification system (Darmstadt, Germany).

### 2.2. Instruments and Conditions

The Waters XEVO TQ-S micro-triple quadrupole mass spectrometer and ACQUITY H-Class UPLC (Waters Corp., Milford, MA, USA) were used to detect SIPI6398. The process involved the use of MassLynx 4.1 software (Waters Corp.) for both data acquisition and device control.

UPLC HSS T3 column (50 mm × 2.1 mm, 1.8 *μ*m) was employed in this work, and the column temperature was set at 40°C. Acetonitrile-water (with 0.1% formic acid) was chosen as the mobile phase. The flow rate was set at 0.4 mL/min. The entire elution process took 4.5 minutes. The detailed elution program is as follows: from 0 to 3.0 min, it increases from 5% to 60% acetonitrile; from 3.0 to 3.5 min, it remains at 60% acetonitrile; from 3.5 to 3.6 min, it decreases from 60% to 5% acetonitrile; and from 3.6 to 4.5 min, it remains at 5% acetonitrile.

**Figure 2:**
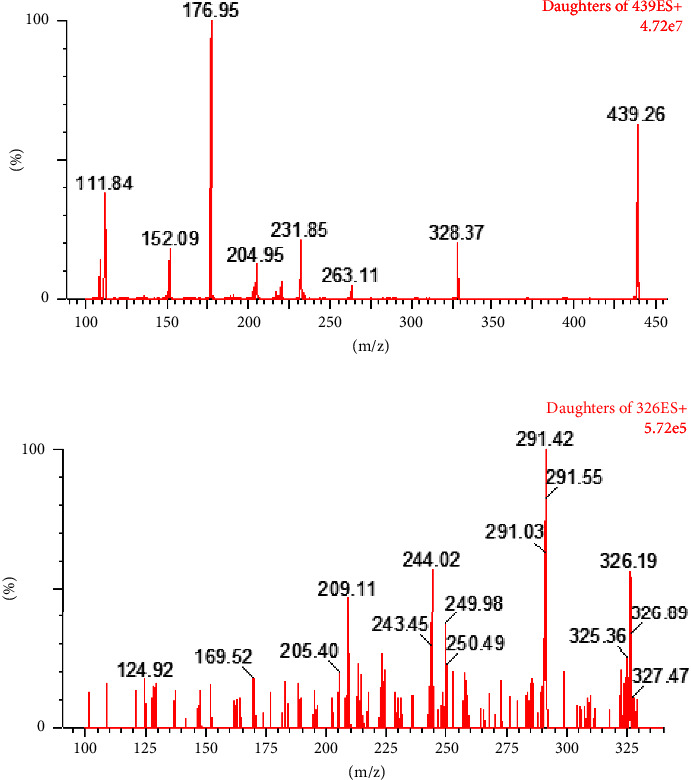
Mass spectrum of SIPI6398 (a) and IS (b).

The cone gas employed was nitrogen, flowing at a rate of 50 L/h, while the desolvation gas utilized was nitrogen, with a flow rate of 993 L/h. The capillary voltage was set to 3.96 kV, the ion source temperature was maintained at 148°C, and the desolvation temperature was optimized to 498°C. Quantitative analysis was conducted using MRM mode in ESI with positive detection, specifically, m/z 439.30 ⟶ 176.90 for SIPI6398. For this analyte, the cone voltage and collision voltage were set to 30 V and 20 V, respectively. Additionally, m/z 326.20 ⟶ 291.40 served as the internal standard with a cone voltage of 30 V and a collision voltage of 32 V (Figure 2).

### 2.3. Standard Curve

SIPI6398 and midazolam were each dissolved in methanol to prepare a stock solution with a concentration of 500 *μ*g/mL. To obtain a series of SIPI6398 concentration standard working solutions of 20, 50, 100, 200, 500, 1000, 2000, 5000, 10000, and 20000 ng/mL, the reserve solution was diluted with methanol. Dilute the stock solution with acetonitrile to prepare a working solution of midazolam at a concentration of 50 ng/mL. All reserve and working solutions should be stored at 4°C to ensure stability.

The appropriate amount of SIPI6398 working solution was added to the blank plasma of rats to prepare plasma samples with concentrations of 2, 5, 10, 20, 50, 100, 200, 500, 1000, and 2000 ng/mL. The standard curve was created with a concentration range of 2–2000 ng/mL. At the same time, quality control (QC) samples were prepared using the same method with plasma concentrations of 4, 160, and 1600 ng/mL.

### 2.4. Sample Treatment Procedure

The plasma sample (50 *μ*L) was added to a sterile 1.5 mL Eppendorf tube, followed by the addition of a solution (150 *μ*L) containing the internal standard midazolam (50 ng/ml). The solution consisted of acetonitrile and methanol in a ratio of 9 : 1. After thorough mixing using a vortex mixer for 1.0 min, the mixture was centrifuged at 11063 g for 10 minutes at 4°C. Subsequently, the supernatant (100 *μ*L) was transferred to the inner tube of a sample vial for UPLC-MS/MS analysis. Finally, an injection volume of 2 *μ*L was used.

### 2.5. Pharmacokinetic Studies

Twelve healthy male Sprague Dawley rats weighing between 220 and 250 g were obtained from the Animal Experimental Center of Wenzhou Medical University. These animals were housed in sterile cages with a controlled ambient temperature of 24–26°C, following a rotating light-dark cycle. They had ad libitum access to rodent food and water. Prior to the experiment, the animals underwent an overnight fast but were allowed unrestricted access to water. All experimental procedures adhered to the animal experiment guidelines of Wenzhou Medical University, and the research protocol was approved by the university's animal ethics committee (xmsq2023-0656). The experimental animals were randomly divided into two groups consisting of six rats each. One group received intravenous administration of SIPI6398 at a dose of 2 mg/kg, while the other group received oral administration at a dose of 4 mg/kg SIPI6398. At time points corresponding to 0.0833 h, 0.5 h, 1 h, 2 h, 3 h, 4 h, 6 h, 8 h, and 12 h post-administration, respectively, blood samples (0.3 ml) were collected from the tail vein using heparinized tubes followed by centrifugation at a speed of 11063 g for 10 minutes. The resulting supernatant plasma (100 *µ*L) was then transferred into new 1.5 mL Eppendorf tubes and stored at −20°C until analysis.

The plasma was treated with a simple protein precipitation method using acetonitrile-methanol (9 : 1, v/v). Subsequently, the treated plasma was analyzed by UPLC-MS/MS to determine the concentration of SIPI6398 using a standard curve. The plasma concentration-time curves for each rat were obtained and pharmacokinetic parameters were calculated using DAS software (version 2.0, China Pharmaceutical University), including area under the concentration-time curve (AUC), mean residence time (MRT), half-life (*t*_1/2_), volume of distribution (V), clearance (CL), and peak plasma concentrations (*C*_max_). These parameters were calculated based on a non-compartment model. Absolute bioavailability (%) = 100 × AUCpo × Div/(AUC_iv_ × D_po_), where AUC_iv_ and AUC_PO_ represent the AUC of the drug after intravenous and oral administration, respectively, while D_iv_ and D_po_ represent a single dosage of SIPI6398 for intravenous and oral administration, respectively.

## 3. Results

### 3.1. Selectivity

**Figure 3:**
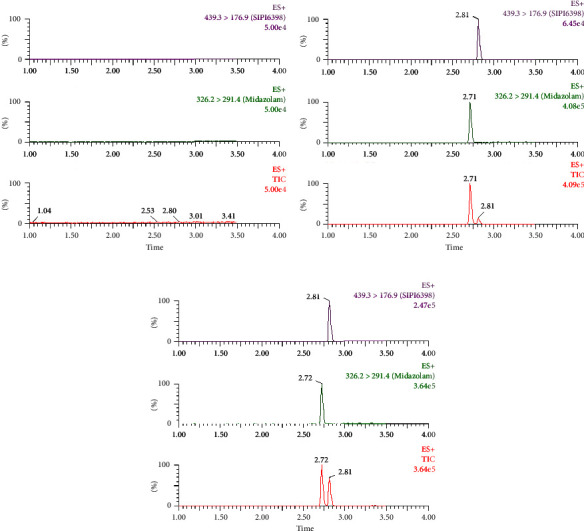
UPLC-MS/MS of SIPI6398 and IS in rat plasma: (a) blank rat plasma; (b) blank rat plasma spiked with LLOQ and IS; (c) rat plasma after intravenous administration of SIPI6398.

According to Figure 3, the retention time of SIPI6398 and the internal standard was determined as 2.81 min and 2.71 min, respectively. By employing an optimized gradient elution program, effective separation of these two substances was achieved without any interference from endogenous components within the observed retention time range of SIPI6398 and the internal standard. Hence, this method exhibits exceptional selectivity.

### 3.2. Standard Curve

The calibration curve of SIPI6398 in rat plasma showed remarkable linearity in the concentration range of 2–2000 ng/mL with an *r* value above 0.99. In rat plasma, the regression equation for SIPI6398 is typically expressed as *y* = 0.00148*x* − 0.02144 (*r* = 0.9990), where *x* represents the concentration of SIPI6398 in plasma and y indicates the ratio of the peak area of SIPI6398 to the internal standard. The lower limit of quantification for SIPI6398 in rat plasma was 2 ng/mL, and the limit of detection was 0.5 ng/mL.

### 3.3. Precision, Accuracy, Recovery, and Matrix Effects

The intra- and inter-day precisions of SIPI6398 were both within 11%, demonstrating excellent reproducibility. The intra- and inter-day accuracies ranged from 90% to 106%, indicating a close agreement between the measured values and true values. Moreover, the recovery of SIPI6398 exceeded 80%, while the matrix effects were observed to be in the range of 89–93%. Detailed data can be found in Table 1.

### 3.4. Stability

The stability of SIPI6398 was assessed through a series of experiments, including pretreatment in the autoinjector for 12 hours, storage at room temperature for 2 hours, three freeze-thaw cycles, and storage at −20°C for 30 days. The results demonstrated that the accuracy of SIPI6398 ranged from 86% to 113%, with a relative standard deviation (RSD) within 13% (Table 2). These findings highlight the exceptional stability exhibited by SIPI6398.

### 3.5. Pharmacokinetics

**Figure 4:**
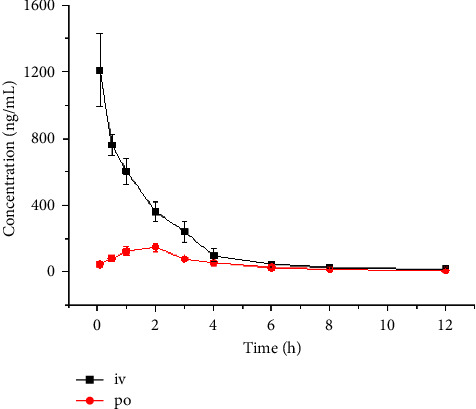
The concentration-time curve of rats after intravenous (i.v., 2 mg/kg) and oral (p.o., 4 mg/kg) administration of SIPI6398.

The concentration-time curve of SIPI6398 in rat plasma is shown in Figure 4. The figure clearly shows the concentration of SIPI6398 in rat plasma at different time points.  Table 3 lists the main pharmacokinetic parameters. AUC_(0-*t*)_ was 2092.8 ± 264.0 ng/mL^*∗*^h and 550.7 ± 39.5 ng/mL^*∗*^h for intravenous (i.v., 2 mg/kg) and oral (p.o., 4 mg/kg) administration of SIPI6398 in rats, respectively. *t*_1/2z_ was 2.6 ± 0.8 h and 2.8 ± 1.2 h; CLz was 0.9 ± 0.1 L/h/kg and 6.9 ± 0.4 L/h/kg; *C*_max_ was 1208.1 ± 218.8 ng/mL and 151.4 ± 26.5 ng/mL for intravenous and oral administration of SIPI6398 in rats, respectively. It should be noted that the oral bioavailability of this compound is low, only 13.2%.

## 4. Discussion

To accurately determine SIPI6398 and internal standard substances based on the chemical structure properties of these two analytes, we employed the positive ESI source mode in conjunction with the MRM mode. The ESI positive ion mode, which is well suited for most organic compounds, was utilized for detection purposes. For quantitative analysis, an MRM mode was adopted to effectively minimize background interference while enhancing detection sensitivity and specificity. Throughout this process, meticulous optimization of parameters such as cone voltage, collision voltage, ion source temperature, nebulization gas flow, and drying gas flow was conducted to enhance the mass spectrometry detection performance of each analyte.

UPLC HSS T3 column (50 mm × 2.1 mm, 1.8 *μ*m) was employed due to its exceptional stability and separation efficacy, rendering it particularly suitable for precise compound separation and analysis. To improve the symmetry of the chromatographic peaks and the retention time of the analytes, the composition and ratio of the mobile phase were studied and optimized based on the selected chromatographic column. After comparing different organic solvents (such as methanol and acetonitrile) and continuously adjusting the gradient program, we found that using an acetonitrile-water system as a mobile phase can make the retention time of each analyte more stable. In addition, to further improve the symmetry and detection sensitivity of chromatographic peaks, we considered the addition of formic acid to improve the peak shape and response value based on the chemical properties of the analyte. After comparing different volume fractions (0.1% and 0.5%) of formic acid, we found that 0.1% formic acid not only effectively improved the peak shape but also significantly improved the detection sensitivity. Therefore, we ultimately decided on 0.1% formic acid as the optimal modifier.

When selecting an internal standard, due to the difficulty of obtaining a homologue of SIPI6398, we tried to use midazolam, which has a similar molecular weight and structure to SIPI6398. After verification, we found that midazolam had similar retention behavior to SIPI6398, with moderate retention time, good peak shape, and high extraction yield. Therefore, we believe that midazolam is an appropriate internal standard.

The pharmacokinetics of SIPI6398, including plasma and brain, were measured in rats [7]. The half-life of the brain samples was >15 hours regardless of intravenous or oral administration. The half-life of the plasma samples was 0.8 hours and 1.7 hours for intravenous and oral administration. SIPI6398 demonstrated high brain penetration with a brain-plasma AUC ratio of 16.1 : 1 despite low oral bioavailability (13.0%) in rats. While the half-life of the plasma samples in this study was 2.6 ± 0.8 hours and 2.8 ± 1.2 hours when administered intravenously and orally, it was not the same as reported. The oral bioavailability of SIPI6398 was 13.2% in this study and was consistent with the literature [7].

## 5. Conclusion

Through the development of UPLC-MS/MS technology, this study successfully determined the accurate measurement of SIPI6398 in rat plasma. After rigorous validation, the method showed excellent performance in terms of accuracy, precision, specificity, and linear response. The pharmacokinetic properties of SIPI6398 in rats and relevant bioavailability data were determined. In the future, the pharmacokinetic properties of SIPI6398 will be studied in detail in other animal models and humans to provide more reliable evidence for optimal drug design and clinical application.

## Figures and Tables

**Table 1:** Accuracy, precision, matrix effect, and recovery of SIPI6398 in rat plasma.

Concentration (ng/mL)	Accuracy (%)		Precision (%RSD)		Matrix effect (%)	Recovery (%)
	Intra-day	Inter-day	Intra-day	Inter-day		
4	96.2	104.0	10.8	8.2	89.4	80.7
160	99.0	90.5	4.3	9.6	92.2	80.9
1600	104.7	105.5	1.0	5.1	92.6	83.3

**Table 2:** Stability of SIPI6398 in rat plasma.

Concentration (ng/mL)	Autosampler (4°C, 12 h)		Ambient (2 h)		−20°C (30 d)		Freeze-thaw	
	Accuracy	RSD	Accuracy	RSD	Accuracy	RSD	Accuracy	RSD
4	101.4	6.5	99.1	2.3	98.3	11.5	112.2	12.7
160	97.5	2.2	106.9	9.1	96.5	2.2	86.8	6.7
1600	101.1	4.6	94.1	2.2	105.2	10.1	101.0	3.5

**Table 3:** Main pharmacokinetic parameters after intravenous (i.v., 2 mg/kg) and oral (p.o., 4 mg/kg) administration of SIPI6398 in rats.

Parameters	Unit	i.v.	p.o.
AUC_(0-*t*)_	ng/mL^*∗*^h	2092.8 ± 264.0	550.7 ± 39.5
AUC_(0-*∞*)_	ng/mL^*∗*^h	2130.4 ± 260.4	577.8 ± 33.9
MRT_(0-*t*)_	h	2.0 ± 0.1	3.2 ± 0.2
MRT_(0-*∞*)_	h	2.4 ± 0.3	4.0 ± 0.7
*t*_1/2*z*_	h	2.6 ± 0.8	2.8 ± 1.2
*T*_max_	h	—	1.7 ± 0.5
CLz	L/h/kg	0.9 ± 0.1	6.9 ± 0.4
Vz	L/kg	3.5 ± 1.3	28.2 ± 12.4
*C*_max_	ng/mL	1208.1 ± 218.8	151.4 ± 26.5

## Data Availability

The data used to support the findings of this study are included within the article.
